# Cannings models, population size changes and multiple-merger coalescents

**DOI:** 10.1007/s00285-020-01470-5

**Published:** 2020-02-01

**Authors:** Fabian Freund

**Affiliations:** Crop Plant Biodiversity and Breeding Informatics Group (350b), Institute of Plant Breeding, Seed Science and Population Genetics, University of Hohenheim, Fruwirthstrasse 21, 70599 Stuttgart, Germany

**Keywords:** $\varLambda$-*n*-coalescent, Cannings models, Population size, Moran model, 92D25, 60J27

## Abstract

Multiple-merger coalescents, e.g. $\varLambda$-*n*-coalescents, have been proposed as models of the genealogy of *n* sampled individuals for a range of populations whose genealogical structures are not captured well by Kingman’s *n*-coalescent. $\varLambda$-*n*-coalescents can be seen as the limit process of the discrete genealogies of Cannings models with fixed population size, when time is rescaled and population size $N\rightarrow \infty$. As established for Kingman’s *n*-coalescent, moderate population size fluctuations in the discrete population model should be reflected by a time-change of the limit coalescent. For $\varLambda$-*n*-coalescents, this has been explicitly shown for only a limited subclass of $\varLambda$-*n*-coalescents and exponentially growing populations. This article gives a more general construction of time-changed $\varLambda$-*n*-coalescents as limits of specific Cannings models with rather arbitrary time changes.

## Introduction

The genealogies of samples from populations with highly variant offspring numbers, for instance due to sweepstake reproduction or rapid selection, are not well modelled by Kingman’s *n*-coalescent. As a more realistic alternative, multiple-merger coalescents, especially $\varLambda$-coalescents have been proposed, as reviewed in Tellier and Lemaire (2014), Irwin et al. (2016) and Eldon et al. (2016). $\varLambda$-*n*-coalescents, introduced by Pitman (1999), Sagitov (1999), Donnelly and Kurtz (1999), are Markovian processes $(\varPi _t)_{t\ge 0}$, which describe the genealogy of a set of individuals $\{1,\ldots ,n\}$. This is done by representing the ancestral lineages present at time *t* of these individuals by the sets of offspring of each ancestral lineage in the sample. Thus, $(\varPi _t)_{t\ge 0}$ can be defined as a random process with states in the set of partitions of $\{1,\ldots ,n\}$ and transitions via merging of blocks (i.e. merging of ancestral lineages to a common ancestor). For a $\varLambda$-*n*-coalescent, the infinitesimal rates of any merger of *k* of *b* present lineages is given by $\lambda _{b,k}:=\int _0^1 x^{k-2}(1-x)^{b-k}\varLambda (dx)$, where $\varLambda$ is a finite measure on [0, 1]. This includes Kingman’s *n*-coalescent if $\varLambda$ is the Dirac measure in 0.

As in the case of Kingman’s *n*-coalescent being the limit genealogy from samples taken from a discrete Wright–Fisher or Moran model, $\varLambda$-*n*-coalescents can be constructed as the (weak) limit of genealogies from samples of size *n* taken from Cannings models. The limit is reached as population size *N* goes to infinity and time is rescaled, see Möhle and Sagitov (2001). Time is rescaled by using $[c^{-1}_N]$ generations in the discrete model as one unit of evolutionary (coalescent) time in the limit, where $c_N$ is the probability that two individuals picked in a generation have the same parent one generation before. In the discrete models, the population size *N* is fixed across all generations.

Only populations in an equilibrium state are described well by models with fixed population sizes. This idealized condition often does not apply to natural populations. In particular, due to fluctuating environmental conditions population sizes are expected to fluctuate likewise. Two standard models of population size changes are timespans of exponential growth or decline, as well as population bottlenecks, where population size drops to a fixed size smaller than *N* for a timespan on the evolutionary (coalescent) timescale. Such changes are featured in coalescent simulators as ms (Hudson 2002) or msprime (Kelleher et al. 2016). The latter changes are also the model of population size changes in PSMC (Li and Durbin 2011) or similar approaches as SMC++ (Terhorst et al. 2017). For the Wright–Fisher model, which converges to Kingman’s *n*-coalescent if population size *N* is fixed for all generations, the same scaling $c_N^{-1}$ from discrete genealogy to limit is valid for population size changes which maintain a population size of order *N* at all times, see Griffiths and Tavare (1994) or Kaj and Krone (2003). The resulting limit process is Kingman’s *n*-coalescent, whose timescale is (non-linearly) transformed. However, size changes too extreme can yield a non-bifurcating (multiple merger) genealogy, see Birkner et al. (2009, Sect. 6.1).

For $\varLambda$-*n*-coalescents, the link between fluctuating population sizes in the discrete models and the time-change in the coalescent limit is somewhat less established. While conditions for convergence of the discrete genealogies to a limit process are given in Möhle (2002), no explicit construction of haploid Cannings models leading to an analogous limit, a $\varLambda$-*n*-coalescent with changed time scale, is given. For a specific case, the Dirac *n*-coalescent for an exponentially growing population, such a construction has been given in Matuszewski et al. (2017), based on the fixed-*N* Cannings model (modified Moran model) from Eldon and Wakeley (2006). However, also other $\varLambda$-*n*-coalescents (or Cannings models which should converge to these) with changed time scale have been recently discussed and applied as models of genealogies, see Spence et al. (2016), Kato et al. (2017), Alter and Louzoun (2016) and Hoscheit and Pybus (2019). This leads to the goal of this article, which is to extend the approach in Matuszewski et al. (2017) to explicitly give a construction of time-changed $\varLambda$-coalescents as limits of Cannings models with fluctuating population sizes. The Cannings models used are modified Moran models, see e.g. Huillet and Möhle (2013), and the Cannings models introduced in Schweinsberg (2003). The main tool to establish the convergence to the time-changed $\varLambda$-*n*-coalescent is, as in Matuszewski et al. (2017), applying Möhle (2002, Thm. 2.2).

For diploid Cannings models, the umbrella model from Koskela and Wilke Berenguer (2019) gives a general framework to add population size changes, selection, recombination and population structure to the fixed-*N*-model. There, if one only considers population size changes, the limit is a time-changed $\varXi$-*n*-coalescent, a coalescent process with simultaneous multiple mergers. The focus in the present paper is slightly different though, the aim is to explicitly construct Cannings models that converge, after linear time scaling, to a time-changed $\varLambda$-*n*-coalescent, while Koskela and Wilke Berenguer (2019) concentrates on the convergence itself.

## Models and main results

Cannings models (Cannings 1974, 1975) describe the probabilistic structure of the pedigree (offspring-parent relations) of a finite population in generations $v\in {\mathbb {Z}}=\{\ldots ,-2,-1,0,1,2\ldots \}$ with integer-valued population sizes $(N_v)_{v\in {\mathbb {Z}}}$. The $N_v$ individuals in generation *v* produce $(\nu ^{(v)}_1,\ldots ,\nu ^{(v)}_{N_v})$ offspring, where $\sum _{i=1}^{N_v}\nu ^{(v)}_i=N_{v+1}$ and offspring sizes are exchangeable, i.e. $(\nu ^{(v)}_1,\ldots ,\nu ^{(v)}_{N_v}) {\mathop {=}\limits ^{d}}(\nu ^{(v)}_{\sigma (1)},\ldots ,\nu ^{(v)}_{\sigma (N_v)})$ for any permutation $\sigma \in S_{N_v}$. The offspring generation $v+1$ then consists of these individuals in arbitrary order (independent of the parents). The case $N_v=N$ for all *v* is denoted as the fixed-*N* case.

From now on, look at the genealogy of the population in generation 0. For convenience, denote the generations in reverse order by $r=-v$, i.e. if one looks *i* generations back, this is denoted by $r=i$. The population sizes $N_r$ are defined relative to a reference size *N*, in a way that if $N\rightarrow \infty$, also $N_r\rightarrow \infty$. From now on, use $N=N_0$. The goal is to establish a limit process of the discrete genealogies as $N\rightarrow \infty$. The discrete genealogy of a sample of size *n* in generation 0 is a random process $({\mathcal {R}}^{(N)}_r)_{r\in {\mathbb {N}}_0}$ with values in the partitions of $\{1,\ldots ,n\}$, where *i*, *j* are in the same block of ${\mathcal {R}}^{(N)}_r$ iff they share the same ancestor in generation *r*.

The terminology from Möhle (2002) is used with slight adaptations. Let $c_{N,r}$ be the probability that two arbitrary individuals in generation $r-1$ have the same ancestor in generation *r* in the model with reference population size *N*. To clarify, $c_{N,r}$ is the coalescence probability for individuals in generation $r-1$ if population sizes are variable, while $c_N$ denotes the coalescence probability in the fixed-*N* case. Define $F_{N}(s)=\sum _{r=1}^{\left[ s\right] }c_{N,r}$ and let

1 $$\begin{aligned} {\mathcal {G}}^{-1}_{N}(t)=\inf \left\{ s>0: F_N(s)>t\right\} -1 \end{aligned}$$

be its shifted pseudo-inverse. For *l* and $a_1,\ldots ,a_l\ge 1$, set

2 $$\begin{aligned} \varPhi ^{(N)}_l(r;a_1,\ldots ,a_l)=\frac{(N_r)_lE\left( \prod _{i=1}^l (\nu _i^{(r)})_{a_i}\right) }{(N_{r-1})_{\sum ^l_i a_i}} \end{aligned}$$

as the probability that in generation $r-1$, from $\sum _{i=1}^l a_i$ individuals sampled from the Cannings model, specific sets of $a_1\ge \cdots \ge a_l$ individuals each find a common ancestor one generation before (generation *r*), where ancestors of different sets are different. For $l=1,a_1=2$, $c_{N,r}=\varPhi ^{(N)}_1(r;2)$. See Möhle (1998) for details.

Consider a sequence of fixed-*N* Cannings models for each $N\rightarrow \infty$ with $c_N\rightarrow 0$ as $N\rightarrow \infty$ and transition probabilities $\varPhi ^{(N)}_l(a_1,\ldots ,a_l)$ for a merger of $a_1,\ldots ,a_l\ge 1$ individuals , converging to a $\varLambda$-*n*-coalescent $(\varPi _t)_{t\ge 0}$ with infinitesimal transition rates $\phi _l(a_1,\ldots ,a_l):=\lambda _{\sum _{i=1}^l a_i,a_1}1_{\{a_2=\cdots =a_l=1\}}$ when scaled by $c_N^{-1}$, i.e.

3 $$\begin{aligned} \left( {\mathcal {R}}^{(N)}_{[c_N^{-1}t]}\right) _{t\ge 0}{\mathop {\rightarrow }\limits ^{d}} (\varPi _t)_{t\ge 0} \end{aligned}$$

in the Skorohod sense as $N\rightarrow \infty$. Eq. (3) is satisfied if

$$\begin{aligned} c_N\rightarrow 0 \,\, \text{ and } \,\, c_N^{-1}\varPhi ^{(N)}_l (a_1,\ldots ,a_l)\rightarrow \phi _l(a_1,\ldots ,a_l) \end{aligned}$$

for $a_1\ge \cdots \ge a_l\ge 2$ as $N\rightarrow \infty$, see (Möhle and Sagitov 2001, Thm. 2.1). We will establish a variant of Möhle (2002, Corollary 2.4) to show convergence of a variety of Cannings models with variable population sizes to (time-changed) $\varLambda$-*n*-coalescents. For this, we need some assumptions. Most importantly, an asymptotically infinite sum needs to be controlled. For this, we introduce a concept of *o*-terms: A sequence $(y_N)_{N\in {\mathbb {N}}}$ of null sequences $y_N=(y_N(r))_{r\in {\mathbb {N}}_0}$ is $o_{\sum }(x_N)$ for a null sequence $(x_N)_{N\in {\mathbb {N}}}$ if summing the first $O(x^{-1}_N)$ members of $y_N$ still vanishes as $N\rightarrow \infty$. This will be denoted by $y_N(r)=o_{\sum }(x_N)$, i.e.

$$\begin{aligned} y_N(r)=o_{\sum }(x_N) \Leftrightarrow \sum ^{[tx_{N}^{-1}]}_{r=0} y_N(r) \rightarrow 0 \,\, \text{ as }\,\, N\rightarrow \infty \,\, \text{ for } \text{ any }\,\, t\ge 0. \end{aligned}$$

For instance, this condition is satisfied if $y_N(r)/x_N$ have the same null sequence majorant as $N\rightarrow \infty$ for all $r\in {\mathbb {N}}$.

Fix $t>0$. We assume for all $0\le r \le [c_{N}^{-1}t]$:

- Population size changes of order *N* leading to a well-defined population size profile in coalescent time, i.e.
  4 $$\begin{aligned}&0<N^{-}(t):=c_1(t)N \le N_r \le c_2(t)N=:N^{+}(t)<\infty \nonumber \\&N^{-1} N_{\lfloor tc_N^{-1}\rfloor }\rightarrow \nu (t) \text{ for } N\rightarrow \infty \end{aligned}$$
  for positive and finite functions $c_1,c_2,\nu :{\mathbb {R}}_{\ge 0} \rightarrow {\mathbb {R}}_{>0}$.
- 5 $$\begin{aligned} \varPhi ^{(N)}_l(r;a_1,\ldots ,a_l)=\varPhi ^{(N_r)}_l(a_1,\ldots ,a_l)+o_{\sum }(c_{N}) \end{aligned}$$

The first class of Cannings models used to construct time-changed $\varLambda$-*n*-coalescents are modified Moran models. In a modified Moran model, only a single individual has more than one offspring (and may have many offspring). Following Huillet and Möhle (2013), define the modified (haploid) Moran model with fixed population size *N*. Let $(U_N(z))_{z\in {\mathbb {N}}}$ be i.i.d. random variables with values in $\{2,\ldots ,N\}$, let $U_N$ be a r.v. with their common distribution. In each generation $z\in {\mathbb {Z}}$,

- One randomly chosen individual has $U_N(z)$ offspring,
- $U_N(z)-1$ randomly chosen individuals have no offspring,
- The other $N-U_N(z)$ have one offspring each,

Specific modified Moran models leading to Dirac *n*-coalescents as genealogy limits have been introduced as population models with skewed offspring distributions, see Eldon and Wakeley (2006) and Matuszewski et al. (2017), for fixed and variable population sizes.

For any $\varLambda$-*n*-coalescent with $\varLambda ([0,1])=1$ (denoted by $\varLambda \in {\mathcal {M}}[0,1]$), Möhle (2013, Prop. 3.4) show that there always exist fixed-*N* modified Moran models such that their rescaled genealogies converge to the $\varLambda$-*n*-coalescent. These can be constructed via a random variable $U'_N$, that is distributed like the merger size of the first merger in a $\varLambda$-*N*-coalescent. As shown in (Huillet and Möhle 2013, Eq. 9), this means

6 $$\begin{aligned} P(U'_N=j)=\lambda _N^{-1} \left( {\begin{array}{c}N\\ j\end{array}}\right) E(X^{j-2}(1-X)^{N-j}),\quad j\ge 2, \end{aligned}$$

where $\lambda _N$ is the total transition rate of the $\varLambda$-*N*-coalescent and *X* has distribution $\varLambda$.

To add population size changes from generation to generation to the modified Moran model, the relationship between offspring and parent generation needs to be defined. This will be done by adjusting the fixed-*N* model: If in generation *r*, there are $N_r$ individuals, first run a fixed-$N_r$ modified Moran model, producing $N_r$ (potential) offspring. Let $U_{n,r}$ denote the number of offspring in generation $r-1$ of the multiplying parent in generation *r*.

**Fig. 1:**
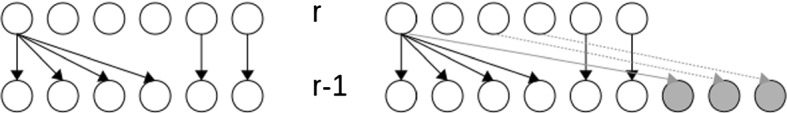
Example of allocation of individuals when population size is increasing. Left: start with a fixed size Moran model with $U_{6}=4$. Right: population increases by $d_{6,r}=3$, from which $A_{n,r}=1$ individual is allocated to the multiplying parent from generation *r* in the fixed size model (and 2 to non-reproducing individuals from the fixed size model in generation *r*). This results in $U_{6,r}=5$

If population size declines from generation *r* to $r-1$, sample $N_{r-1}$ individuals randomly (without replacement) from the $N_{r}$ potential offspring consisting of $U_{N_r}$ offspring of the multiplying parent and $N_{r}-U_{N_r}$ single offspring. If individuals are added to the population (population growth), i.e.

7 $$\begin{aligned} d_{N,r}=N_{r-1}-N_r>0, \end{aligned}$$

to still end up with a modified Moran model one has two options. Additional individuals can be added as further offspring of the already multiplying parent. A second option is to add individuals as offspring of the non-reproducing individuals from generation *r* in the fixed-$N_r$ model. Each originally non-reproducing parent can have one offspring, so this allows one to add $U_{N_r}-1\ge 1$ individuals. The number of additional individuals can be divided between these two options, let $A_{n,r}$ denote the individuals added as offspring to the multiplying parent (which means that $d_{N,r}-A_{N,r}$ individuals are added as offspring of non-reproducing parents from the fixed-$N_r$ model). Expressed differently, there are $N_{r-1}=N_r+d_{N,r}$ offspring from which $U_{n,r}=U_{N_r}+A_{N,r}$ share the same parent, while all other offspring are single offspring of other parents (who all differ). See Fig. 1 for an example.

While some care has to be taken to not change coalescence probabilities (see Remark 5 for an example), there will be different possibilities to choose $A_{N,r}$. For Dirac-*n*-coalescents with exponential growth (on the coalescent time scale), Matuszewski et al. (2017) used $A_{n,r}=d_{N,r}$. A reasonable approach may also be to set $A_{n,r}$ (close to) proportional to the fraction $U_{N_r}/N_r$ of offspring coming from the multiplying parent: Each of the $d_{N,r}$ added individuals are added to the multiplying parent with probability $U_{N_r}/N_r$ (with the obvious constraint that after $U_{N_r}-1$ individuals are added as offspring of non-reproducing parents, all further individuals need to be added to the multiplying parent). As for the fixed-size models, we consider the genealogy of a sample of *n* individuals, which is denoted by $(\tilde{{\mathcal {R}}}^{(N)}_r)_{r\in {\mathbb {N}}_0}$

The main results of the present paper show that the two allocation schemes allow one to construct $\varLambda$-*n*-coalescent limits of the genealogies of these modified Moran models if population sizes vary in the discrete models in ways described by Eq. (4).

### Theorem 1

Let $\varLambda \in {\mathcal {M}}[0,1]$ so that $U'_N$ defined by Eq. (6) satisfies

$$\begin{aligned} E((U'_N)_2)(N-1)^{-1}\nrightarrow 0 \,\, \text{ for } \,\, N\rightarrow \infty . \end{aligned}$$

Define a modified Moran model for fixed *N* by

$$\begin{aligned} U_N:=U'_N1_{A_N}+2(1-1_{A_N}) \end{aligned}$$

for sets $A_N$ s.t. $U'_N$,$1_{A_N}$ are independent and $E((U'_N)_2)P(A_N)((N)_2)^{-1}=N^{-\gamma }$ for $1<\gamma <2$. Let $\nu :{\mathbb {R}}_{\ge 0}\rightarrow {\mathbb {R}}_{>0}$ be a positive real function. Then, there exist population sizes satisfying Eq. (4) for $\nu$ so that the genealogies $(\tilde{{\mathcal {R}}}^{(N)}_r)_{r\in {\mathbb {N}}_0}$ of the modified Moran model with variable population sizes converge

$$\begin{aligned} \left( \tilde{{\mathcal {R}}}^{(N)}_{[c_N^{-1} t]}\right) _{t\ge 0}{\mathop {\rightarrow }\limits ^{d}}(\varPi _{{\mathcal {G}}(t)})_{t\ge 0} \end{aligned}$$

in the Skorohod-sense, where ${\mathcal {G}}(t)=\int ^t_0 (\nu (s))^{-\gamma } ds$ and $(\varPi _t)_{t\ge 0}$ is a $\varLambda$-*n*-coalescent. In the discrete model, additional individuals can be added in any way so that the resulting model is still a modified Moran model.

For $\varLambda$ not covered by Theorem 1, one can choose slightly different modified Moran models that converge to a $\varLambda$-*n*-coalescent limit for an arbitrary population size profile on the coalescent time scale.

### Theorem 2

Fix $\varLambda \in {\mathcal {M}}[0,1]$ so that $U'_N$ defined by Eq. (6) satisfies

$$\begin{aligned} E((U'_N)_2)(N-1)^{-1}\rightarrow 0. \end{aligned}$$

For fixed population size *N*, define modified Moran models via $U_N=U'_N$. Let $\nu :{\mathbb {R}}_{\ge 0}\rightarrow {\mathbb {R}}_{>0}$ be a positive function describing the population size profile. Then, there exist population sizes satisfying Eq. (4) for $\nu$ so that the genealogies $(\tilde{{\mathcal {R}}}^{(N)}_r)_{r\in {\mathbb {N}}_0}$ of the modified Moran model with variable population sizes fulfill $(\tilde{{\mathcal {R}}}^{(N)}_{[{\mathcal {G}}_N^{-1}(t)]})_{t\ge 0}{\mathop {\rightarrow }\limits ^{d}}(\varPi _t)_{t\ge 0}$ in the Skorohod-sense, where $(\varPi _t)_{t\ge 0}$ is a $\varLambda$-*n*-coalescent. In the discrete model, additional individuals are added solely as offspring of non-reproducing parents from the fixed-$N_r$ model, unless $E((U_N)_2)\rightarrow \infty$ as $N\rightarrow \infty$. In that case, they can be added any way that preserves that the model is still a modified Moran model.

### Remark 1

The condition of $\varLambda ([0,1])=1$ in both theorems is not very important: If one scales by $c_2c_N$ instead of $c_N$ for any $c_2>0$, the rescaled discrete genealogies converge to the $c_2\varLambda$-*n*-coalescent.

To get convergence to a time-changed $\varLambda$-*n*-coalescent in Theorem 2, i.e.

$$\begin{aligned} \left( \tilde{{\mathcal {R}}}^{(N)}_{[c_N^{-1} t]}\right) _{t\ge 0}{\mathop {\rightarrow }\limits ^{d}}(\varPi _{{\mathcal {G}}(t)})_{t\ge 0} \end{aligned}$$

in the Skorohod-sense as $N\rightarrow \infty$, one needs that

8 $$\begin{aligned} {\mathcal {G}}(t):= \lim _{N\rightarrow \infty }\sum _{r=0}^{[tc_N^{-1}]}c_{N_r} \end{aligned}$$

exists for all $t\ge 0$. The following corollary shows that, at least for certain measures $\varLambda$, this condition is met.

### Corollary 1

Let $\varLambda \in {\mathcal {M}}[0,1]$ be a Beta(a,b)-distribution with $a\in (0,1)$ and $b>0$. Let $\nu :{\mathbb {R}}_{\ge 0}\rightarrow {\mathbb {R}}_{>0}$. Then, there exist population sizes satisfying Eq. (4) for $\nu$ so that the genealogies $(\tilde{{\mathcal {R}}}^{(N)}_r)_{r\in {\mathbb {N}}_0}$ of the modified Moran model with variable population sizes fulfill $(\tilde{{\mathcal {R}}}^{(N)}_{[ c_N^{-1}t]})_{t\ge 0}{\mathop {\rightarrow }\limits ^{d}}(\varPi _{{\mathcal {G}}(t)})_{t\ge 0}$ in the Skorohod-sense, where ${\mathcal {G}}(t)=\int ^t_0 (\nu (s))^{a-2} ds$ and $(\varPi _t)_{t\ge 0}$ is a *Beta*(*a*, *b*)-*n*-coalescent. In the discrete model, additional individuals can be added in any way so that the resulting model is still a modified Moran model.

The specific models used in each of the two theorems are not the only possibilities of modified Moran models with variable population sizes to converge to $\varLambda$-*n*-coalescents. For instance, if one only allows certain population size changes, one can also use the modified Moran model used in Theorem 2 for some $\varLambda$ covered by Theorem 1.

### Corollary 2

Let $\varLambda \in {\mathcal {M}}[0,1]$ be a Beta(a,b)-distribution with $a\in (1,2)$ and $b>0$. Consider an exponentially growing modified Moran model population on the coalescent time scale, i.e. $\nu (t)=\exp (-\rho t)$ Then, there exist population sizes satisfying Eq. (4) for $\nu$ so that the genealogies $(\tilde{{\mathcal {R}}}^{(N)}_r)_{r\in {\mathbb {N}}_0}$ of the modified Moran model with variable population sizes fulfills $(\tilde{{\mathcal {R}}}^{(N)}_{[c_N^{-1}t]})_{t\ge 0}{\mathop {\rightarrow }\limits ^{d}}(\varPi _{{\mathcal {G}}(t)})_{t\ge 0}$ in the Skorohod-sense, where ${\mathcal {G}}(t)=\int ^t_0 (\nu (s))^{a-2} ds$ and $(\varPi _t)_{t\ge 0}$ is a $\varLambda$-*n*-coalescent. In the discrete model, additional individuals can be added in any way so that the resulting model is still a modified Moran model.

Finally, for the classic Moran model, i.e. the modified Moran model with $U_N=U'_N$ and $U'_N$ defined via Eq. (17) for $\varLambda =\delta _0$, we can establish

### Proposition 1

For the standard Moran model and a population size profile $\nu :{\mathbb {R}}_{\ge 0}\rightarrow {\mathbb {R}}_{>0}$, there exist population size changes allowed by Eq. (4) so that $(\tilde{{\mathcal {R}}}^{(N)}_{[c_N^{-1}t]})_{t\ge 0}{\mathop {\rightarrow }\limits ^{d}}(\varPi _{{\mathcal {G}}(t)})_{t\ge 0}$ in the Skorohod-sense, where ${\mathcal {G}}(t)=\int ^t_0 (\nu (s))^{-2} ds$. Individuals are added only as offspring of non-reproducing parents (in the fixed-$N_r$ model) if the population size increases.

For Beta$(2-\alpha ,\alpha )$-*n*-coalescents, $1\le \alpha <2$, genealogies sampled from the fixed-*N* Cannings models introduced in Schweinsberg (2003) also converge weakly to these Beta coalescent processes (after rescaling of time) as $N\rightarrow \infty$.

This model (for fixed population size *N*) lets each individual in any generation *r* produce a number of (potential) offspring $X^{(r)}_i$, i.i.d. across individuals and generations, distributed as a tail-heavy random variable *X* with $E(X)=\mu >1$, i.e.

9 $$\begin{aligned} P(X\ge k)\sim Ck^{-\alpha }\,\, \text{ on } \,\, {\mathbb {N}}, \end{aligned}$$

where $C>0$ is a constant and $1<\alpha <2$. Then, *N* offspring are chosen to form the next generation. If less than *N* offspring are produced, the missing next generation individuals are arbitrarily associated with parents. Here, this is done by randomly choosing a parent, which preserves exchangeability and makes the model a Cannings model. The genealogies of a sample of size *n* converge as $N\rightarrow \infty$ and time rescaled by $c_N^{-1}$ to the Beta($2-\alpha$,$\alpha$)-*n*-coalescent, see Schweinsberg (2003, Thm. 4).

This model can very easily extended to variable population sizes by just sampling from the potential offspring. The tail-heavy distributions used produce, asymptotically as $N\rightarrow \infty$, enough potential offspring to cover growing population sizes of order *N* as allowed by Eq. (4).

### Lemma 1

Let $d_{N,r}:=N_{r-1}-N_r>0$. Assume that for any fixed *t*, for all $r\le c^{-1}_Nt$ there exists a null sequence $(d_N)_{N\in {\mathbb {N}}}$ with $d_{N,r}/N\le d_N$ as $N\rightarrow \infty$. Then, $P(\sum _{i=1}^{N_r} X^{(r)}_{i}<N_{r-1})\le A^{N}$ with $N=N_0$ and $A<1$.

This gives us an alternative Cannings model with variable population sizes to define time-changed Beta coalescents as the limit of its discrete genealogies.

### Theorem 3

Consider the Cannings model coming from sampling from potential i.i.d. offspring following Eq. (9) with parameter $\alpha \in [1,2)$. For any $\nu :{\mathbb {R}}_{\ge 0}\rightarrow {\mathbb {R}}_{>0}$, there exist variable population sizes $(N_r)_{r\in {\mathbb {N}}_0}$ fulfilling (4) for $\nu$ so that the discrete *n*-coalescents converge $(\tilde{{\mathcal {R}}}^{(N)}_{[c_N^{-1}t]})_{t\ge 0}{\mathop {\rightarrow }\limits ^{d}}(\varPi _{{\mathcal {G}}(t)})_{t\ge 0}$ in the Skorohod-sense, where ${\mathcal {G}}(t)=\int ^t_0 (\nu (s))^{1-\alpha } ds$ and where $(\varPi _t)_{t\ge 0}$ is the Beta($2-\alpha ,\alpha$)-*n*-coalescent.

The time-change function ${\mathcal {G}}(t)$, which appears in Theorem 1, Corollary 2, Propositions 1 and 3 simplifies considerably for exponential growth on the coalescent time scale, i.e. $\nu (t)=exp(-\rho t)$ for $\rho >0$ in Eq. (4) (corresponding to population sizes given by $N_{r-1}=\lfloor N_r(1-c_N\rho )\rfloor$ for $r\in {\mathbb {N}}$).

### Corollary 3

For a population size profiles of exponential growth (on the coalescent scale) with growth rate $\rho$ and for $c_N=cN^{-\gamma }+o(N^{-\gamma })$ for $\gamma >0$, the time-change function ${\mathcal {G}}$ has the form

10 $$\begin{aligned} {\mathcal {G}}(t)=\int _0^t e^{\rho \gamma s}ds=(\rho \gamma )^{-1}(e^{\rho \gamma t}-1). \end{aligned}$$

This implies that the waiting time between coalescent events are Gompertz distributed with parameters $a=\lambda _be^{\rho \gamma t_0}$ and $b=\rho \gamma$, i.e. the waiting time *T* for the next coalescence event, given the last coalescence at $t_0$ into *b* lineages, fulfills

$$\begin{aligned} P_{t_0}(T\le t)&=1-\exp (\lambda _b(\rho \gamma )^{-1} (e^{\rho \gamma (t+t_0)}-e^{\rho \gamma t_0}))\\&=1-\exp (\lambda _b(\rho \gamma )^{-1}e^{\rho \gamma t_0}/e^{\rho \gamma t}-1). \end{aligned}$$

### Remark 2

It is well-known that for Kingman’s *n*-coalescent with exponential growth, waiting times for coalescence events follow a Gompertz distribution, e.g. see Slatkin and Hudson (1991, Eq. 5), Polanski et al. (2003). For time-changed Dirac coalescents appearing as limits of modified Moran models with $\nu (t)=exp(-\rho t)$, Eq. (10) appeared in Matuszewski et al. (2017).

## Discussion

As for the Wright–Fisher model, genealogies of samples taken from (haploid) modified Moran and other Cannings models can be approximated by a time-change of their limit coalescent process, when the population sizes of the discrete models are fluctuating, but are always of the same order of size. As for models with fixed population size, time intervals of $[c^{-1}_N t]$ generations in the discrete model correspond to a time interval of length *t* in the continuous time limit. The approach of this study was to build on existing Cannings models that converge for fixed population size to the $\varLambda$-*n*-coalescent and just change the population sizes gradually from generation to generation, which includes adjusting parent-offspring allocation between generations. This raises the question whether the used Cannings models and the adjustment of ancestral relationships have biological interpretations and are a reasonable model for at least some real populations.

### Interpretation of the Cannings models and allocation schemes used

The modified Moran models used to construct a time-changed $\varLambda$-*n*-coalescent with $\varLambda ([0,1])=1$ [defined via Eq. (6), introduced in Huillet and Möhle (2013)] can be described as follows (for fixed *N*): On top of a standard Moran model choice of one parent *M* with two offspring and one individual in the parent generation with no offpring, there is a random probability *X* for each other individual in the parent generation to not have offspring in the next generation. *X* is drawn from $\varLambda$, potentially only activated in a given generation with a low probability $N^{-\gamma }$, $\gamma \in (1,2)$. From the individuals that have offspring, all but *M* reproduce once, and *M* replaces itself and all non-reproducing individuals by its offspring. These models capture the concept of sweepstake reproduction (Hedgecock and Pudovkin 2011), though the assumption of a single individual with more than one offspring is rather artificial. For a non-random *X* and large families appearing occasionally at rate of order $N^{-\gamma }$, this model is very similar to the discrete modified Moran model from Eldon and Wakeley (2006) used to describe sweepstake reproduction (and that was used in Matuszewski et al. (2017) as a basis to construct a time-changed Dirac *n*-coalescent). Both models lead to the same Dirac coalescent limit and have the same time rescaling order $c_N^{-1}$. In Eldon and Wakeley (2006), instead of randomly choosing individuals to not reproduce with probability *X*, a fixed number of $\approx NX-2$ individuals are chosen at random to not reproduce on top of the Moran choice (again with a small probability in each generation for this to happen). For random *X*, similar models also appear in Hartmann and Huillet (2018) and Eldon (2012).

The other class of Cannings models used to capture skewed offspring distributions, defined via Eq. (9), lead to the specific class of Beta($2-\alpha$, $\alpha$)-*n*-coalescents. They have been proposed as a model of type-III survivorship, where all individuals produce many offspring with a high juvenile mortality, see e.g. Steinrücken et al. (2013, Sect. 2.3), also leading to sweepstake-like phenomena. While both classes of Cannings models allow the Bolthausen-Sznitman *n*-coalescent ($\varLambda =\hbox {Beta}(1,1)$) as a possible limit model, the discrete models used to explicitly construct it are not based on modelling a directed selection process due to selective advantages of certain ancestral lineages. Thus, the results do not answer whether adding population size changes to a model of rapid selection or genetic draft as in Desai et al. (2013), Neher and Hallatschek (2013), Schweinsberg (2017) also leads to its rescaled genealogies being described by a time-changed Bolthausen-Sznitman *n*-coalescent.

To construct time-changed $\varLambda$-*n*-coalescents as limits of genealogies in modified Moran models, the approach here is to adjust fixed-*N* modified Moran models for growing or decreasing population sizes. Sampling the next generation from the fixed-*N* offspring when there is population decline maintains on average the ratio between the large family $U_N$ and the rest off the individuals. This means that the population decrease, e.g. due to less resources available, has the same chance to affect each offspring of the fixed-size model. Additional individuals can be added to the family of the multiplying parent or by allowing parents with no offspring from the fixed-*N* allocation scheme to have exactly one offspring. For some sequences of modified Moran models, any partition of additional individuals to these two allocation forms is possible, e.g. allocate them randomly to the multiplying parent (with $U_{N_r}$ offspring) from the fixed-size model with probability $U_{N_r}/N_r$ (with the constraint that we cannot add more than $U_{N_r}-1$ individuals to non-reproducing parents). The merit of this random allocation is that it is trying to maintain the ratio $U_{N_r}/N_r$ from the fixed-size model. As for sampling a smaller number of individuals, this describes that population size increase, e.g. due to more resources available, follows (approximately and on average) the sweepstake pattern of the fixed-*N* model. From a biological viewpoint, other allocation schemes can also be interpreted: Adding the additional offspring completely to the largest family, as done in Matuszewski et al. (2017), could describe a scenario where new resources become available and only the multiple-offspring parent can claim them for its offspring. In contrast, adding individuals as single offspring of non-reproducing parents from the fixed-size model relaxes the (viability) “selection” pressure of the modified Moran model by allowing more non-multiplying parents (resp. their offspring) to survive, e.g. due to the additional resources. For the models covered in Theorem 3 from Schweinsberg (2003), population size changes in either direction are modelled by sampling from a pool of more individuals than the current population size, thus additional or decreasing resources affect the offspring of different parents in the same way.

### Influence of the choice of Cannings model on the limit

Many results in the present paper allow us to scale the time in the discrete models with $c_N^{-1}$ as in the fixed *N* case so that the scaled genealogies converge to a time-changed $\varLambda$-*n*-coalescent $(\varPi _{{\mathcal {G}}(t)})_{t\ge 0}$. This time-change ${\mathcal {G}}(t)$ depends both on the population size profile $\nu$ on the coalescent time scale from Eq. (4) and the (asymptotic properties of) the coalescence probabilities $c_N$, i.e. how many discrete generation correspond to one unit of coalescent time. For instance, consider an exponentially growing population (on the coalescent time scale, $\nu (t)=\exp (-\rho t)$ for $\rho > 0$) and two different models leading to a time-changed $Beta(2-\alpha ,\alpha )$-*n*-coalescent ($\alpha \in (1,2)$): the ones from Corollary 1 and Theorem 3. From Eq. (10), we see that ${\mathcal {G}}$ depends on the product $\gamma \rho$. For the model from Corollary 2, $\gamma =\alpha$ and for the one from Proposition 3, it is $\gamma =\alpha -1$. Thus, the exact same time-changed $\varLambda$-*n*-coalescent can appear as limit model for genealogies with different population size profiles on the coalescent time scale. As already discussed in (Matuszewski et al. 2017) in the case of time-changed Dirac-*n*-coalescents, this poses a problem for inference: If one wants to infer $\rho$ directly (instead of the compound parameter $\gamma \rho$), $\gamma$ has to be known. This means that specifying/identifying the Cannings model leading to the limit process would be necessary to directly estimate $\rho$. This is very similar to the effect that e.g. Watterson’s estimator only estimates the mutation rate on the coalescent time scale, and not the mutation rate in one generation, see e.g. Eldon and Wakeley (2006, p. 2627). Another example for different $\nu$ leading to the same time-scaled coalescent limit for different Cannings models is given by the genealogy limit from the Wright–Fisher model and the (usual) Moran models. It is well known, see e.g. Griffiths and Tavare (1994), that the rescaled genealogy of a sample from a Wright–Fisher model with population size profile $\nu$ converges to Kingman’s *n*-coalescent with time change ${\mathcal {G}}$ as in Eq. (22) with $\gamma =1$. However, for the classic Moran model, Proposition 1 shows that Eq. (22) holds with $\gamma =2$.

For families of Cannings models, if the coalescence probability $c_N$ is of order $\log (N)^{-1}$, a curious phenomenon appears: Population size changes of order *N* do not even alter the limit genealogy. An example is the model from Proposition 3 for the Bolthausen-Sznitman *n*-coalescent ($\varLambda =Beta(1,1)$). One can interpret this for a population described by the model as follows: Even instantaneous bottlenecks or expansions do not influence the effect that a very large family appearing in a generation has on the genealogy. How the population reproduces, i.e. how the offspring distributions compare between different parents, is thus fully controlling the genealogy, regardless of changes that alter the population sizes overall, e.g. changes in range and/or resources.

## Proofs

This section contains the proof of the presented statements as well as some further remarks.

### Converging to a time-changed coalescent: sufficient conditions

First, recall this special case of Möhle (2002, Thm. 2.2)

#### Corollary 4

If we satisfy, for any fixed t,

11 $$\begin{aligned}&\lim _{N\rightarrow \infty } \inf _{1\le r\le {\mathcal {G}}^{-1}_{N}(t)} N_r =\infty ,\quad \lim _{N\rightarrow \infty } \sup _{1\le r \le {\mathcal {G}}^{-1}_{N}(t)} c_{N,r}=0, \end{aligned}$$

12 $$\begin{aligned}&\lim _{N\rightarrow \infty } \sum _{r=1}^{{\mathcal {G}}^{-1}_{N}(t)} \varPhi ^{(N)}_l(r;a_1,\ldots ,a_l)=q_{a_1,\ldots ,a_l}t<\infty , a_1 \ge \cdots \ge a_l\ge 2 \end{aligned}$$

the discrete-time coalescent $(\tilde{{\mathcal {R}}}^{(N)}_{[{\mathcal {G}}_N^{-1}(t)]})_{t\ge 0}$, so rescaled in time, converges in distribution (Skorohod-sense) to a continuous-time Markov chain with transition function $\exp ({Qt})$, where *Q* is a transition rate matrix with entries $q_{a_1,\ldots ,a_l}$, $a_1\ge \cdots \ge a_l\ge 2$ (so diagonal entries are the negative row sums of the other entries).

#### Remark 3

When compared to the original formulation of Möhle (2002, Thm 2.2), the limit here can be described as a homogeneous Markov chain with rate matrix *Q* instead of the more complicated original description of the transition probabilities as a product integral of matrix-valued measures. This directly follows from the stronger condition (12), where for Möhle (2002, Thm 2.2) to hold only convergence and not linear dependence on *t* is needed. Indeed, if (12) holds, the value $\varPi ((0,t])$ of the product measure $\varPi$ in Möhle (2002, Thm. 2.2) has the form *Qt*. This is stated on Möhle (2002, p. 209), see also Eq. (24) therein. Then, the form of the transition function is described on Möhle (2002, p. 203).

Now, recall the conditions (4), (5). Additionally, consider the following control condition for the fluctuations of $c_N$ as $N\rightarrow \infty$:

For $t>0$, there exist $M_1(t),M_2(t)\in (0,\infty )$ with

13 $$\begin{aligned} M_1(t)\le \frac{c_{N_r}}{c_N}\le M_2(t) \end{aligned}$$

for all $r\le [t c_N^{-1}]$. For instance, when (4) holds, this condition is satisfied if $c_N=f(N)$, where *f* is regularly varying (at $\infty$). If Eqs. (5) and (13) hold, choosing $l=1$ in Eq. (5) yields

14 $$\begin{aligned} M_1(t)c_{N} + o_{\sum }(c_N) \le c_{N,r} \le M_2(t)c_{N} + o_{\sum }(c_N) \end{aligned}$$

as $$N\rightarrow \infty $$.

Now, we establish an easy-to-verify variant of Möhle (2002, Corollary 2.4).

#### Lemma 2

Consider a sequence of Cannings models with reference size $N=N_0$ and variable population size $(N_r)_{r\ge 0}$ which fulfill conditions (4), (5), (13), $\lim _{N\rightarrow \infty }c_N = 0$ and whose genealogies of a sample of size *n*, if one would fix the population sizes $N_r\equiv N_0$, would be in the domain of attraction of a $\varLambda$-*n*-coalescent $(\varPi _t)_{t\ge 0}$ (rescaled by $c_N^{-1}$). Then, Corollary 4 can be applied, so $(\tilde{{\mathcal {R}}}^{(N)}_{[{\mathcal {G}}_N^{-1}(t)]})_{t\ge 0}{\mathop {\rightarrow }\limits ^{d}}(\varPi _t)_{t\ge 0}$ in the Skorohod-sense.

If furthermore ${\mathcal {G}}^{-1}(t):=\lim _{N\rightarrow \infty } {\mathcal {G}}_N^{-1}(t)c_N$ exists, we have, with ${\mathcal {G}}=({\mathcal {G}}^{-1})^{-1}$,

15 $$\begin{aligned} \left( \tilde{{\mathcal {R}}}^{(N)}_{[t/c_N]}\right) _{t\ge 0}{\mathop {\rightarrow }\limits ^{d}} (\varPi _{{\mathcal {G}}(t)})_{t\ge 0} \end{aligned}$$

as $$N\rightarrow \infty $$

#### Proof

Size changes of order *N* satisfy the first part of Condition (11). Its second part is then satisfied by (14), which in turn is satisfied due to (5) and (13). Also due to (14), $F_N$ is bounded by

16 $$\begin{aligned} {[}s]M_1(t')c_N+[s]o(c_N)\le F_{N}(s)\le [s]M_2(t')c_N+[s]o(c_N) \end{aligned}$$

as $N\rightarrow \infty$ and $[s]\le c_{N}^{-1}t'$ and thus its pseudo-inverse by

$$\begin{aligned} \frac{t}{M_2(t')c_N}+\frac{o(c_N)}{c_N}-1 \le {\mathcal {G}}_N^{-1}(t) \le \frac{t}{M_1(t')c_N}+\frac{o(c_N)}{c_N}-1 \end{aligned}$$

with an appropriate $t'\ge t$. This implies that the time change function ${\mathcal {G}}_N^{-1}$ for the discrete models in Corollary 4 is of order $c^{-1}_N$. Knowing this, we compute

$$\begin{aligned} \sum _{r=1}^{{\mathcal {G}}^{-1}_{N}(t)} \varPhi ^{(N)}_l(r;a_1,\ldots ,a_l)&{\mathop {=}\limits ^{(5)}} \sum _{r=1}^{{\mathcal {G}}^{-1}_{N} (t)}\underbrace{\varPhi ^{(N_r)}_l(a_1,\ldots ,a_l)c_{N_r}^{-1}}_{\rightarrow \phi _l(a_1,\ldots ,a_l)}c_{N_r}+\sum _{r=1}^{{\mathcal {G}}^{-1}_{N}(t)} o_{\sum }(c_N) \\&{\mathop {=}\limits ^{(5)}}\phi _l(a_1,\ldots ,a_l) \underbrace{\sum _{r=1}^{{\mathcal {G}}^{-1}_{N}(t)}c_{N,r}}_{=F_N({\mathcal {G}}^{-1}_{N} (t))}+ O(1)\underbrace{\sum _{r=1}^{{\mathcal {G}}^{-1}_{N}(t)} o_{\sum }(c_N)}_{\rightarrow 0} \\&\rightarrow \phi _l(a_1,\ldots ,a_l)t \end{aligned}$$

as $N\rightarrow \infty$ The second equation is valid due to the uniform convergence of

$\varPhi ^{(N_r)}_l(a_1,\ldots ,a_l)c_{N_r}^{-1}$ in *r* for $N\rightarrow \infty$ ($N_r$ is bounded from below on the timescale used). This allows us to pull out $\phi _l(a_1,\ldots ,a_l)$. This shows that condition 12 is satisfied and thus establishes the convergence of $(\tilde{{\mathcal {R}}}^{(N)}_{[{\mathcal {G}}_N^{-1} (t)]})_{t\ge 0}$ to the same $\varLambda$-*n*-coalescent as the fixed-size model. Eq. (15) follows as described in Möhle (1998, Sec. 4). $\square$

#### Remark 4

The condition for Eq. (15) to hold is a weak condition, since ${\mathcal {G}}_N^{-1}(t)$ is of order $c^{-1}_N$. Additionally, the linear scaling in (15) makes it easy to introduce a mutation structure. Let mutation be introduced in the discrete model by allowing mutations from parent to offspring with a rate $\mu _N$. If $\mu _Nc_N^{-1}\rightarrow \theta$ as $N\rightarrow \infty$, the mutations on the time-scaled $\varLambda$-*n*-coalescent are given by a Poisson point process with homogeneous intensity $\theta$.

The next step is to establish a special case of Lemma 2 which only considers modified Moran models with changing population sizes.

#### Remark 5

Depending on the magnitude of a population size increase, adding individuals as further offspring of the multiplying parent from the fixed-size modified Moran model can strongly increase coalescence probabilities. For instance, for a population expansion of size *Nm*, if one just expands by adding $d_{N,r}=Nm$ to the offspring number of the individual with multiple offspring in a single generation, the coalescence probability for this generation is dominated by the population size change. Then $U_{N,r}\ge Nm$, leading to $c_{N,r}=\frac{E((U_{N,r})_2)}{(N_{r-1})_2}\ge \frac{(Nm-1)^2}{(N_{r-1})_2}=O(1)\nrightarrow 0$ as $N\rightarrow \infty$. Thus, from generation $r-1$ to *r*, coalescence is still happening with positive probability as $N\rightarrow \infty$, which shows that a potential limit coalescent cannot just be a (non-degenerately) time-changed $\varLambda$-*n*-coalescent, a continuous-time (inhomogeneous) Markovian process. This has an implication for modelling of real populations: The genealogy of a sudden population expansion, happening at a specific generation, where a single genotype/individual is responsible for the population growth, is not given by a time-changed continuous-time $\varLambda$-*n*-coalescent.

We recall some properties of fixed-*N* modified Moran models.

#### Lemma 3

(i) As $N\rightarrow \infty$: $U_N/N{\mathop {\rightarrow }\limits ^{d}}0$ is equivalent to $c_N=\frac{E((U_N)_2)}{(N)_2}\rightarrow 0$
(ii) If $c_N\rightarrow 0$ as $N\rightarrow \infty$, the genealogies in the modified Moran models converge, with a rescaling of time by $c_N^{-1}$, to a $\varLambda$-*n* coalescent if
  17 $$\begin{aligned} \lim _{N\rightarrow \infty }c_N^{-1}\varPhi ^{(N)}_l(a_1,\ldots ,a_l) =\lim _{N\rightarrow \infty }1_{\{l=1\}}\frac{E((U)_{a_1})}{(N)_{a_1}c_N} =\int ^1_0 x^{a_1-2}\varLambda (dx)1_{\{l=1\}} \end{aligned}$$
(iii) If $U'_N$ is distributed as in Eq. (6)
  18 $$\begin{aligned} E((U'_N)_k)=\frac{(N)_k}{\lambda _N}E(X^{k-2}) \end{aligned}$$
  for all $$k\ge 2$$.

#### Proof

(i) from Huillet and Möhle (2013, Lemma 3.2), (ii) from Huillet and Möhle (2013, Theorem 3.3), (iii) from Huillet and Möhle (2013, Eq. 10) $\square$

The following proposition provides criteria for genealogies in modified Moran models with fluctuating population sizes to converge to a $\varLambda$-*n*-coalescent after a suitable time change.

#### Proposition 2

Consider a fixed-*N* modified Moran model so that $U_N/N{\mathop {\rightarrow }\limits ^{d}} 0$ as $N\rightarrow \infty$ and that (17) holds for a finite measure $\varLambda$ on [0, 1]. From this, construct a modified Moran model with varying population sizes $(N_r)_{r\ge 0}$ which satisfy the following conditions. Assume that Eqs. (4) and (13) are satisfied. Assume further $d_{N,r}/N_r\le d_N\rightarrow 0$ as $N\rightarrow \infty$. Let $A_{n,r}$ be the number of individuals in generation $r-1$ allocated as offspring of the multiplying parent of the fixed-$N_r$ model from generation *r*. If $P(A_{N,r}>0)>0$, further assume $\frac{E(U_N)}{E((U_N)_2)}\rightarrow 0$ and $A_{N,r}\le c_4E(U_N)$ for a constant $c_4>0$ and $N\rightarrow \infty$. Additionally, assume $d_{N,r}-A_{n,r}\le \min \{i:P(U_{N_r}=i)>0\}-1$.

Based on the fixed-size modified Moran model and $(N_r)_{r\in {\mathbb {N}}}$ define a modified Moran model with population sizes $(N_r)_{r\in {\mathbb {N}}}$ and offspring variable $U_{N,r}=U_{N_r}+A_{N,r}$ for all $r\in {\mathbb {N}}$.

Then, $(\tilde{{\mathcal {R}}}^{(N)}_{[{\mathcal {G}}_N^{-1}(t)]})_{t\ge 0}{\mathop {\rightarrow }\limits ^{d}}(\varPi _t)_{t\ge 0}$ in the Skorohod-sense, where $(\varPi _t)_{t\ge 0}$ is the $\varLambda$-*n*-coalescent limit for the fixed-*N* modified Moran model.

#### Proof

This is shown by applying Lemma 2. All conditions but Eq. (5) of it are clearly fulfilled under the assumptions of the proposition currently proven, see also Lemma 3.

To show (5), first assume $d_{N,r}\ge 0$. Then,

19 $$\begin{aligned} \frac{E((U_{N_r}+A_{N,r})_{a_1})}{(N_r)_{a_1}c_N}&=\sum _{k=1}^{a_1}s(a_1,k)\sum _{l=0}^k\left( {\begin{array}{c}k\\ l\end{array}}\right) \frac{E(U_{N_r}^lA_{N,r}^{k-l})}{(N_r)_{a_1}c_N}\nonumber \\&=\frac{E((U_{N_r})_{a_1})}{(N_r)_{a_1}c_N} +\sum _{k=1}^{a_1}s(a_1,k)\sum _{l=0}^{k-1}\left( {\begin{array}{c}k\\ l\end{array}}\right) \frac{E(U_{N_r}^lA_{N,r}^{k-l})}{(N_r)_{a_1}c_N}, \end{aligned}$$

where *s*(*n*, *k*) are Stirling numbers of the first kind. From Eq. (17), we see that $\frac{E((U_{N_r})_{a_1})}{(N_r)_{a_1}c_N}\rightarrow \int _0^1 x^{k-2}\varLambda (dx)$ uniformly in *r* as $N\rightarrow \infty$ (convergence of the first summand is at least as fast as for $N^-(t)$), while we will now show that the sum following this term in Eq. (19) vanishes asymptotically. We will give an upper bound for $\frac{E(U_{N_r}^lA_{N,r}^{k-l})}{(N_r)_{a_1} c_N}$, independent from *r*. For this, we need to recall several technical points: For an upper bound, we can always omit terms of the form $\frac{U_M}{M}\le 1$ for $M\in {\mathbb {N}}$, we assume $A_{N,r}\le c_4E(U_N)$ for $N\in {\mathbb {N}}$ and $E(U_N)(E((U_N)_2))^{-1}\rightarrow 0$ as $N\rightarrow \infty$ in this proposition and we have

20 $$\begin{aligned} \frac{N_r^{a_1}}{(N_r)_{a_1}}\le \frac{a_1^{a_1}}{a_1!}, \end{aligned}$$

which follows from the fact that $x\mapsto \frac{x^{a_1}}{(x)_{a_1}}$ decreases for $x\ge a_1$. With all this, we can observe that, for $0\le r \le c_{N}^{-1}t$ and $0\le l< k<a_1$,

$$\begin{aligned} 0&\le \frac{E(U_{N_r}^lA_{N,r}^{k-l})}{(N_r)_{a_1}c_N} \le \frac{E(U^l_{N_r})c_4^{k-l}(E(U_N))^{k-l}N_r^{a_1}}{N_r^{a_1}c_N (N_r)_{a_1}} {\mathop {\le }\limits ^{(4)}} \frac{c_4^{k-l}E(U_{N})^{k-l} N_r^{a_1}}{(c_1(t))^{a_1-l}N^{a_1-l} c_N (N_r)_{a_1}}\\&{\mathop {\le }\limits ^{(20)}} \frac{c_4^{k-l}E(U_N)a_1^{a_1}}{(c_1(t))^{a_1-l}N^2c_N a_1!} \le \frac{c_4^{k-l}(N)_2E(U_N)a_1^{a_1}}{(c_1(t))^{a_1-l}N^2E((U_N)_2)a_1!} \rightarrow 0 \end{aligned}$$

as $N\rightarrow \infty$. We can thus establish convergence, uniform in *r*, in Eq. (19), since we have just shown the uniform convergence of all its summands:

21 $$\begin{aligned} \frac{E((U_{N_r}+A_{N,r})_{a_1})}{(N_r)_{a_1}c_N} \rightarrow \int _0^1 x^{k-2}\varLambda (dx) \end{aligned}$$

as $$N\rightarrow \infty $$.

Regardless of the allocation of the new individuals, the population model is a modified Moran model with a single multiplying parent. Thus, to show Eq. (5) one only needs to show $\varPhi _1^{(N)}(r;a_1)=\varPhi _1^{(N_r)}(a_1)+o_{\sum }(c_N)$ for $0\le r \le c_{N}^{-1}t$. Compute further

$$\begin{aligned}&\varPhi ^{(N)}_l(r;a_1)=E((U_{N_r}+A_{N,r})_{a_1})/(N_r+d_{N,r})_{a_1}\\&\quad = \frac{(N_r)_{a_1}}{(N_r+d_{N,r})_{a_1}}E((U_{N_r}+A_{N,r})_{a_1})/(N_r)_{a_1}\\&\quad {\mathop {=}\limits ^{(*)}}(1-o(1))^{-1}\left( \frac{E((U_{N_r})_{a_1})}{(N_r)_{a_1}} +o_{\sum }(c_N)\right) =\varPhi ^{(N_r)}_1(a_1)+o_{\sum }(c_N). \end{aligned}$$

Equation $(*)$ follows from Eq. (21) and, for the first factor, from $N^{-1}d_{N,r}\le d_N$ being a null sequence.

Now, consider $d_{N,r}<0$. Then, we get the offspring population by sampling $N_{r-1}$ individuals out of $N_r$, from which $U_{N_r}$ share one common parent. Thus, this is again a modified Moran model, where $U_{n,r}$ is conditionally hypergeometrically distributed with $$P(U_{N,r}=k|U_{N_r}) =\frac{\left( {\begin{array}{c}U_{N_r}\\ k\end{array}}\right) \left( {\begin{array}{c}N_r-U_{N_r}\\ N_{r-1}-k\end{array}}\right) }{\left( {\begin{array}{c}N_r\\ N_{r-1}\end{array}}\right) }$$. Using the factorial moment of the hypergeometric distribution leads to

$$\begin{aligned} \varPhi ^{(N)}_1(r;a_1)&= E\left( \frac{(U_{n,r})_{a_1}}{(N_{r-1})_{a_1}}\right) =\frac{E(E((U_{n,r})_{a_1}\vert U_{N_r}))}{(N_{r-1})_{a_1}} =\frac{(N_{r-1})_{a_1}}{(N_{r-1})_{a_1}}\frac{E((U_{N_r})_{a_1})}{(N_r)_{a_1}}\\&=\varPhi ^{(N_r)}(a_1). \end{aligned}$$

$$\square $$

#### Remark 6

In Proposition 2, if additionally ${\mathcal {G}}^{-1}(t):=\lim _{N\rightarrow \infty } {\mathcal {G}}_N^{-1}(t)c_N$ exists for all $t\ge 0$, Eq. (15) is satisfied, too (since Lemma 2 holds).

Finally, the following lemma provides sufficient conditions for shifting the time-change ${\mathcal {G}}_N^{-1}$ from pre-limit to limit.

#### Lemma 4

Assume that for a Cannings model with variable population sizes $(N_r)_{r\in {\mathbb {N}}}$, the discrete *n*-coalescents satisfy $(\tilde{{\mathcal {R}}}^{(N)}_{[{\mathcal {G}}_N^{-1}(t)]})_{t\ge 0}{\mathop {\rightarrow }\limits ^{d}}(\varPi _t)_{t\ge 0}$ in the Skorohod-sense as $N\rightarrow \infty$, where $(\varPi _t)_{t\ge 0}$ is a $\varLambda$-*n*-coalescent and ${\mathcal {G}}^{-1}_N$ is defined via Eq. (1). Further assume that Eq. (4) is satisfied for a positive real function $\nu$ and that $c_N=f(N)+o_{\sum }(c_N)$ for a function $f(x)=cx^{-\gamma }$ for $\gamma >0$ or $f(x)=c\log (x)^{-1}$ for a constant $c>0$. Then, the convergence can be equivalently expressed as $({\mathcal {R}}_{c_N^{-1}t})_{t\ge 0}{\mathop {\rightarrow }\limits ^{d}} (\varPi _{{\mathcal {G}}(t)})_{t\ge 0}$ in the Skorohod-sense, where ${\mathcal {G}}(t)=\int ^t_0 (\nu (s))^{-\gamma } ds$, where $\gamma =0$ is used if $f(x)=c\log (x)^{-1}$.

#### Proof

${\mathcal {G}}$ is the pseudo-inverse of $\lim _{N\rightarrow \infty }{\mathcal {G}}^{-1}_Nc_N$, so

22 $$\begin{aligned} {\mathcal {G}}(t)=\lim _{N\rightarrow \infty } F_N(tc_{N}^{-1}), \end{aligned}$$

since for a sequence of functions, the inverses converge iff the original functions converge and since *cf* has the inverse $t\mapsto f^{-1}(c^{-1}t)$. The shift by -1 does not alter the limit here, since its effect vanishes as $N\rightarrow \infty$ due to the multiplication with $c_N$. It is important to note here that any terms of order $o_{\sum }(c_N)$ can be omitted when computing $F_N$. Thus, we can replace $c_{N,r}$ by $c_{N_r}$ and even by $c^*_{N_r}=f(N_r)$ for a constant $c_2$. Since $N^{-1}N_{\lfloor tc_N^{-1}\rfloor }\rightarrow \nu (t)$, analogous to Griffiths and Tavare (1994), we can show, for $f(x)=cx^{-\gamma }$,

$$\begin{aligned} {\mathcal {G}}(t)&=\lim _{N\rightarrow \infty }\sum _{r=1}^{[tc_{N}^{-1}]} \frac{c^*_{N_r}}{c^*_N}c^*_N=\lim _{N\rightarrow \infty } \sum _{r=1}^{[tc_{N}^{-1}]} \left( \frac{N}{N_r}\right) ^{\gamma }c^*_N\\&=\lim _{N\rightarrow \infty }\int _0^t \sum _{r=1}^{[tc_N^{-1}]} \left( \frac{N_r}{N}\right) ^{-\gamma }1_{[rc_N,(r+1)c_N)}(s) ds =\int _0^t (\nu (s))^{-\gamma } ds, \end{aligned}$$

where for convergence, observe that there is pointwise convergence

$$\begin{aligned} \sum _{r=1}^{[tc_N^{-1}]} \left( \frac{N_r}{N}\right) ^{-\gamma }1_{[rc_N, (r+1)c_N)}(s)= \left( \frac{N_{\lfloor sc_N^{-1}\rfloor }}{N}\right) ^{-\gamma } \rightarrow (v(s))^{-\gamma } \end{aligned}$$

for $s\in [0,t]$ inside the integral and that bounded convergence is applicable since Eq. (4) ensures that the integrand is in $[M_2(t)^{-\gamma },M_1(t)^{-\gamma }]$. If *f*(*x*) is a logarithm, we have, using $k_r$ defined by $N_r=Nk_r$ for $0\le r \le c_{N}^{-1}t$ with $c_1(t)\le k_r\le c_2(t)$,

$$\begin{aligned} {\mathcal {G}}(t)&=\lim _{N\rightarrow \infty }\sum _{r=1}^{[tc_{N}^{-1}]}c \log (N)^{-1}\frac{\log (N)}{\log (N_r)}\\&=\lim _{N\rightarrow \infty }c \sum _{r=1}^{[tc\log (N)]}\log (N)^{-1} \left( 1-\frac{\log (k_r)}{\log (N)+\log (k_r)}\right) \\&= t-\lim _{N\rightarrow \infty }c\sum _{r=1}^{[tc\log (N)]} \frac{\log (k_r)}{(\log (N)+\log (k_r))\log (N)}=t=\int _0^t (\nu (s))^0 ds. \end{aligned}$$

$$\square $$

#### Remark 7

- The integral representation of the time change is a deterministic version of the coalescent intensity from Kaj and Krone (2003, Sect. 1.3), just applied to Cannings models leading to non-Kingman $\varLambda$-*n*-coalescents.
- As described in Möhle (1998, Section 4), the time-changed $\varLambda$-*n*-coalescent limit can also be expressed by its infinitesimal rates
  $$\begin{aligned} \lambda ^{(\nu )}_{n,k}=(\nu (s))^{-\gamma }\int ^1_0 x^{k-2} (1-x)^{n-k}\varLambda (dx) \end{aligned}$$
  for a merger of *k* of *n* present lineages. This is also the form in which the limit process of the diploid umbrella model from Koskela and Wilke Berenguer (2019) is given.
- Conditioned that the limit coalescent $(\varPi _{{\mathcal {G}}(t)})_{t\ge 0}$ has at time $T_0=t_0$ coalesced into a state with *b* blocks, what is the distribution of the waiting time *T* for the next coalescence event? If $T=t$, this means that in the non-rescaled $\varLambda$-*n*-coalescent $(\varPi _t)_{t\ge 0}$, we wait ${\mathcal {G}}(t)-{\mathcal {G}}(t_0)$ for the next coalescence. This waiting time $T'$ in $(\varPi _t)_{t\ge 0}$ is exponentially distributed with parameter $\lambda _b$ (total rate of coalescence). Thus,
  23 $$\begin{aligned} P(T>t_0+t|T_0=t_0)=P(T'>{\mathcal {G}}(t_0+t)-{\mathcal {G}}(t_0)) =e^{-\lambda _b({\mathcal {G}}(t_0+t)-{\mathcal {G}}(t_0))}.\qquad \end{aligned}$$

#### Proof of Corollary 3

The form of ${\mathcal {G}}$ is a direct consequence of Lemma 4, since $\nu (t)=exp(-\rho t)$. Then, plugging ${\mathcal {G}}$ into Eq. (23) yields the distribution for the next coalescence event, the Gompertz distribution parameters as e.g. described in Lenart (2014, Eq. 3) can be read off. $\square$

### Proofs of convergence to a time-changed coalescent: modified Moran models

The modified Moran models used in Theorems 1 and 2 were introduced in Huillet and Möhle (2013, Prop. 4), the latter model with a small modification to ensure that there is always a parent with at least two offspring, see also Huillet and Möhle (2013, Example 4.1).

#### Proof of Theorem 1

Assume that $E((U'_N)_2)(N-1)^{-1}\nrightarrow 0$ as $N\rightarrow \infty$ also holds for any subsequence. If not, restrict to a subsequence for which this is true and define the limit only along this subsequence.

First, we verify that $c_N=N^{-\gamma }+o_{\sum }(N^{-\gamma })$, thus converges to 0 and that the fixed-*N* model converges to the $\varLambda$-*n*-coalescent. Let $c'_N$ be the coalescence probability in a fixed-*N* modified Moran model with $U'_N$ as the number of offspring of the multiplying parent. This ensures $2((N)_2)^{-1} \le c'_N\le 1$. Moreover, the assumptions made ensure that $(Nc_N')_{N\in {\mathbb {N}}}$ has a lower bound $>0$, so we can define $A_n$ s.t. $P(A_N)c'_N=N^{-\gamma }$ for any $\gamma \in (1,2)$. Then, the following is satisfied as $N\rightarrow \infty$ and $X{\mathop {=}\limits ^{d}}\varLambda$

24 $$\begin{aligned} c_N&=c'_NP(A_N)+(1-P(A_N))\frac{2}{N(N-1)}=N^{-\gamma }+o(N^{-\gamma }),\nonumber \\ \frac{E((U_N)_k)}{(N)_k c_N}&= \frac{E((U'_N)_k)P(A_N)}{(N)_k c_N} {\mathop {=}\limits ^{(*)}}\frac{E((U'_N)_k)N^{-\gamma }\lambda _N}{(N)_kc_N}\nonumber \\&{\mathop {=}\limits ^{(18)}} \frac{E(X^{k-2})N^{-\gamma }}{c_N} \rightarrow E(X^{k-2}) \text{ for } k>3, \end{aligned}$$

where Eq. $(*)$ uses $c'_N=\lambda _N^{-1}$, which follows from Eq. (18) with $k=2$. This establishes the convergence to the $\varLambda$-*n*-coalescent in the fixed-*N* case. Now we assume variable population sizes $(N_r)_{r\in {\mathbb {N}}}$. First, observe that, since $c_N=O(N^{-\gamma })$, it is enough to add occasionally a single individual from generation *r* to $r-1$ to generate any population size changes allowed in Eq. (4) including bottlenecks which are instantaneous on the coalescent time scale. This single individual can then be added as offspring of a non-multiplying parent from the fixed-$N_r$ model or as an offspring of the already multiplying parent. To see the latter, observe that $E((U_N)_2)=c_N(N)_2\sim N^{-\gamma }N^2\rightarrow \infty$. Then, as in Huillet and Möhle (2013, third remark p. 8), one has

$$\begin{aligned} \frac{E(U_N)}{E((U_N)_2)}\sim \frac{E(U_N)}{E(U_N^2)}\le \frac{1}{E(U_N)}. \end{aligned}$$

If both $E((U_N)_2),E(U_N)\rightarrow \infty$, the equation above shows that $\frac{E(U_N)}{E((U_N)_2)}\rightarrow 0$. If $E(U_N)\nrightarrow \infty$ but $E((U_N)_2)$ does, we still have $\frac{E(U_N)}{E((U_N)_2)}\rightarrow 0$ as $N\rightarrow \infty$. Thus, Proposition 1 allows one to add the one individual also to the already multiplying parent.

Thus, we have verified all conditions but Eq. (13) to apply Proposition 2. However, this follows from $c_N$ regularly varying. Since $c_N=N^{-\gamma }+o(N^{-\gamma })$, we can also shift the non-linear time-change to the limit due to Lemma 4. $\square$

For the proof of the next theorem, we use the following

#### Lemma 5

Let $\varLambda \in {\mathcal {M}}[0,1]$ and let $U'_N$ be distributed as in Eq. (6) for any $N\in {\mathbb {N}}$. Let $(N_r)_{r\in {\mathbb {N}}}$ satisfy Eq. (4). Then, Eq. (13) is satisfied.

#### Proof

Equation (18) shows $c_N=\lambda _N^{-1}$, where $\lambda _N$ is the total transition rate for the first jump of a $\varLambda$-*N*-coalescent. Without restriction, assume $N_r\ge N$ (for $N<N_r$, an analogous proof provides bounds for $\frac{c_N}{c_{N_r}}$). Further assume that the *N*-coalescent is just the restriction of the $N_r$-coalescent on individuals $\{1,\ldots ,N\}$. Any merger in the *N*-coalescent is then also a merger in the $N_r$-coalescent, which shows $\lambda _N\le \lambda _{N_r}$. In contrast, the first merger in the $N_r$-coalescent is only a merger in the *N*-coalescent if it features at least two individuals from $\{1,\ldots ,N\}$. The probability of this is bounded from below by the probability $\frac{N(N-1)}{N_r(N_r-1)}$ that the first two of the blocks merged in the *N* are from $\{1,\ldots ,N\}$. This implies

$$\begin{aligned} 0.5(c_2(t))^{-2}{\mathop {<}\limits ^{(4)}} \frac{N(N-1)}{N_r(N_r-1)}\le \frac{c_{N}}{c_{N_r}} =\frac{\lambda _N}{\lambda _{N_r}} \le 1 \end{aligned}$$

#### Proof of Theorem 2

In the fixed-*N* case, Eq. (6) implies that $E((U'_N)_2)(N-1)^{-1}\rightarrow 0$ necessarily needs that $\lambda _N\rightarrow \infty$ as $N\rightarrow \infty$. This is equivalent to $\int _0^1 x^{-2}\varLambda (dx)=\infty$, see (Pitman 1999, Eq. 7). Thus, convergence to the $\varLambda$-*n*-coalescent is shown in (Huillet and Möhle 2013, Prop. 3.4). Now, switch to variable population sizes $(N_r)_{r\in {\mathbb {N}}}$. Since $Nc_N=E((U_N)_2) (N-1)^{-1}\rightarrow 0$ as $N\rightarrow \infty$, it is enough to add one individual per generation to cover any population growth profile covered by Eq. (4). This can always be done by letting a parent not reproducing in the fixed-size model reproduce (once). To add as further offspring of the multiplying parent, assume $E((U_N)_2)\rightarrow \infty$ as $N\rightarrow \infty$. Then, $\frac{E(U_N)}{E((U_N)_2)}\rightarrow 0$ as shown in the proof of Theorem 1. Thus, Proposition 1 provides that at most $A_{N,r}\le c_4E(U_N)$ for arbitrary $c_4>0$ individuals can be added per generation to the already multiplying parent. This allows for adding up to any fixed number $k\in {\mathbb {N}}$ individuals per generation. Additionally, Eq. (13) is satisfied due to Lemma 5. We can thus apply Proposition 2, with an arbitrary allocation of additional individuals that yields a modified Moran model. $\square$

*Proof of: if Theorem*2*holds, Eq.* (8) *implies Eq.* (15) We just need to show that the condition in Remark 6 is equivalent to Eq. (8). From the proof of Lemma 4 [the arguments surrounding Eq. (22)] combined with Eq. (5), we see that existence of $\lim _{N\rightarrow \infty }\sum _{r=0}^{[tc_N^{-1}]}c_{N_r}$ is equivalent to the existence of ${\mathcal {G}}^{-1}(t):=\lim _{N\rightarrow \infty } {\mathcal {G}}_N^{-1}(t)c_N$. $\square$

To prove the Corollaries 1 and 2, we collect some properties of the modified Moran models with $U_N=U'_N$ with $U'_N$ given by Eq. (6) leading to Beta-(*a*, *b*)-coalescents for $a\in (0,2]$, $b>0$. From Huillet and Möhle (2013, Eq. (10) +  Corollary A.1),

25 $$\begin{aligned} c_N\sim \frac{(2-a)\varGamma (b)}{\varGamma (a+b)}N^{a-2} \text{ for } a<2. \end{aligned}$$

#### Proof of Corollary 1

From Eq. (25), it follows that for $a\in (0,1)$, $\varLambda =Beta(a,b)$ satisfies $E((U'_N)_2)(N-1)^{-1}=Nc_N=O(N^{a-1}) \rightarrow 0$ as $N\rightarrow \infty$. Thus, such $\varLambda$-*n*-coalescents are covered by Theorem 2. Additionally from Eq. (25), $c_N$ has a form that is covered by Lemma 4, which allows us to shift the time-change ${\mathcal {G}}$ in Theorem 2 to the limit coalescent and also shows the form of ${\mathcal {G}}$ in Corollary 1. $\square$

#### Proof of Corollary 2

We reiterate the proof of Theorem 2. Let $\varLambda =Beta(a,b)$ for $a\in (1,2)$, which satisfies $\int x^{-2}\varLambda (dx)=\infty$. Thus, in the fixed-*N* case, again (Huillet and Möhle 2013, Prop. 3.4) ensures the convergence of the discrete genealogies to the $\varLambda$-*n*-coalescent when properly rescaled as $N\rightarrow \infty$. Furthermore, Lemma 5 shows that Eq. (13) is satisfied Since $\nu (t)=exp(-\rho t)$, we can use $N_r=\lfloor N(1-\rho c_N)\rfloor$ to satisfy (4). Thus, we only need to show that the population size increase per generation does not violate the conditions of Proposition 2. Indeed,

$$\begin{aligned} d_{N,r}&=\lfloor N( 1-c_N\rho )^{r}\rfloor -\lfloor N( 1-\rho c_N)^{r+1}\rfloor \le N\left( 1 - \left( 1-\rho c_N\right) \right) +1\\&= N(\rho c_N)+1 \end{aligned}$$

individuals at most have to be added. These can be added as $A_{n,r}$ additional offspring of the multiplying parent from the fixed-$N_r$ model, if the condition to apply it from Lemma 2 are met. For $\varLambda$ considered here, one has $E((U_N)_2))\sim N^2 c_N\sim N^{a}\rightarrow \infty$, see Eq. (25). From Lemma 2 we see that then we are allowed to add $O(E(U_N))$ individuals. Huillet and Möhle (2013, Remark p. 9) shows $E(U_N)=c_5Nc_N$ for a constant $c_5>0$, so such growth is indeed covered (and we can then still use $A_{N,r}<d_{N_r}$ and add the other individuals to non-reproducing parents from the fixed-$N_r$ model). Thus, we can establish convergence using Proposition 2 and shift the time-change ${\mathcal {G}}$ to the limit using Lemma 4, since $c_N$ is essentially a negative power of *N*. $\square$

#### Proof of Proposition 1

For $\varLambda =\delta _0$, Eq. (6) shows $U'\equiv 2$, so the modified Moran model is the normal Model model in this case. Since $E((U'_N)_2)(N-1)^{-1}=2(N-1)^{-1}\rightarrow 0$ as $N\rightarrow \infty$, Theorem 2 applies. Since $c_N=2(N(N-1))^{-1}=2N^{-2} + o(N^{-2})$, we can apply Lemma 4 to shift the time-change ${\mathcal {G}}$ to the coalescent limit. $\square$

### Proofs of converging to a time-changed coalescent: model from Schweinsberg (2003)

#### Proof of Lemma 1

It suffices to reiterate the proof of Schweinsberg (2003, Lemma 5) briefly. For $u\in [0,1]$, consider the generating function $f(u):=E(u^X)$. Let $d'_{n,r}=d_{N,r}/N^r$. Then, $S_{N,r}:=\sum _{i=1}^{N_r} X^{(r)}_i$ fulfills

$$\begin{aligned} P(S_{N,r}\le N_{r-1})\le u^{-N_r(1+d'_{N,r})}E(u^{S_{N,r}}) =(u^{-(1+d'_{N,r})}f(u))^{N_r}. \end{aligned}$$

Since $f(1)=1$ and $f'(1)=\mu >1$, there exists $u_0\in (0,1)$ and $\epsilon >0$ so that $u_0^{1+\epsilon }>f(u_0)$. Moreover, for this $\epsilon$ we find $N_0\in {\mathbb {N}}$ so that $d_N<\epsilon$ for $N\ge N_0$. For such *N*, as computed above, one gets

$$\begin{aligned} P(S_{N,r}\le N_{r-1})\le (u_0^{-(1+d'_{N,r})}f(u_0))^{N_r} \le A_1^{N_r} \le A_1^{N^{-}(t)}=(\underbrace{A_1^{c^{-}(t)}}_{<1})^N, \end{aligned}$$

where $A_1:=u_0^{-(1+\epsilon )}f(u_0)<1$. Setting $A:=A_1^{c^{-}(t)}$ completes the proof. $\square$

#### Proof of Theorem 3

Recall that, for $1<\alpha <2$, the coalescence probability in this (fixed-*N*) model satisfies $c_N\sim C\alpha B(2-\alpha ,\alpha )E(X)^{-\alpha }N^{1-\alpha }$, where *B* is the Beta function, see Schweinsberg (2003, Lemma 13). For $\alpha =1$, instead $c_N\sim (\log N)^{-1}$, see Schweinsberg (2003, Lemma 16). Check the conditions necessary to apply Lemma 2: the model and the assumptions above satisfy $c_N\rightarrow 0$ as $N\rightarrow \infty$, (4) and, since $c_N$ is regularly varying, also (13). The changes of population sizes from generation to generation are enough to cover instantanous population size changes on the coalescent time scale (and these are the most extreme changes allowed in Eq. (4)): for a (coalescent time) instantaneous change of size *mN*, one can set $|d_{N,r}|=m\sqrt{c_N}=:d_N\rightarrow 0$ as $N\rightarrow \infty$ for $(\sqrt{c_N})^{-1}$ generations. Thus, only (5) needs to be verified. Since $N_{r-1}$ offspring are sampled from $\sum _{i=1}^{N_r}X^{(r)}_{i}$ potential offspring, the transition probabilities of the discrete coalescent can be formulated analogously to Eq. (2) as

$$\begin{aligned} \varPhi ^{(N)}(r;a_1,\ldots ,a_l)=\frac{(N_r)_lE \left( \prod _{i=1}^l (X^{(r)}_i)_{a_i}\right) }{(N_{r-1})_{\sum ^l_{i=1} a_i}} =\varPhi ^{(N_r)}(a_1,\ldots ,a_l)\frac{(N_{r})_{\sum ^l_{i=1} a_i}}{(N_{r-1})_{\sum ^l_{i=1} a_i}} \end{aligned}$$

This means one just needs to show that

$$\begin{aligned} \varPhi ^{(N_r)}(a_1,\ldots ,a_l)\left| \frac{(N_r)_{\sum _{i=1}^l a_i}}{(N_{r-1})_{\sum _{i=1}^l a_i}}-1\right| =o_{\sum }(c_N), \end{aligned}$$

which follows from

$$\begin{aligned} c_N^{-1}\varPhi ^{(N_r)}(a_1,\ldots ,a_l)\left| \frac{(N_r)_{\sum _{i=1}^l a_i}}{(N_{r-1})_{\sum _{i=1}^l a_i}}-1\right| \rightarrow 0 \end{aligned}$$

uniformly in *r*. To show the latter, uniform convergence, proceed as follows. First, observe that, since (13) holds,

$$\begin{aligned} c_N^{-1}\varPhi ^{(N_r)}(a_1,\ldots ,a_l)=\underbrace{c_{N_r}^{-1} \varPhi ^{(N_r)}(a_1,\ldots ,a_l)}_{\rightarrow \phi (a_1,\ldots ,a_l)}\frac{c_{N_r}}{c_N} \end{aligned}$$

is uniformly bounded (again, since $N_r$ is bounded from below by $N^-(t)$, there is uniform convergence in *r* of the first factor as $N\rightarrow \infty$). Thus, we only need to show $\left| \frac{(N_r)_{\sum _{i=1}^l a_i}}{(N_{r-1})_{\sum _{i=1}^l a_i}}-1\right| \rightarrow 0$. For this, observe that the function $x\mapsto \frac{a'_1-x}{a'_2-x}$ for $x<a'_2$ is strictly increasing (decreasing) if $a'_1-a'_2>0$ (if $a'_1-a'_2<0$). This shows that there are $b_1,b_2\in {\mathbb {N}}_0$ so that

$$\begin{aligned} \left( \frac{N_r-b_1}{N_{r-1}-b_1}\right) ^{\sum _{i=1}^l a_i} \le \frac{(N_r)_{\sum _{i=1}^l a_i}}{(N_{r-1})_{\sum _{i=1}^l a_i}} \le \left( \frac{N_r-b_2}{N_{r-1}-b_2}\right) ^{\sum _{i=1}^l a_i}. \end{aligned}$$

This implies that it is sufficient to show $\left| \left( \frac{N_r-b}{N_{r-1}-b}\right) ^{\sum _{i=1}^l a_i}-1\right| \rightarrow 0$ as $N\rightarrow \infty$ for any $N^-(t)>b\ge 0$, which follows from $\left| \left( \frac{N_r-b}{N_{r-1}-b}\right) -1\right| \rightarrow 0$ uniformly in *r*. Further computation shows

$$\begin{aligned} \left| \frac{N_r-b}{N_{r-1}-b}-1\right| =\frac{|d_{N,r}|}{N_{r-1}-b} \le \frac{d_N}{N_{r-1}-b}. \end{aligned}$$

Since $d_N\rightarrow 0$, this vanishes uniformly. Thus, Lemma 2 can be applied, establishing convergence of $(\tilde{{\mathcal {R}}}^{(N)}_{[{\mathcal {G}}_N^{-1} (t)]})_{t\ge 0}{\mathop {\rightarrow }\limits ^{d}}(\varPi _{t})_{t\ge 0}$. Lemma 4 then ensures that the time-change ${\mathcal {G}}$ can be shifted to the limit, since $c_N$ is either essentially a negative power or a logarithm of *N*. $\square$
